# Application of Antifreeze Substances in Food Cryopreservation

**DOI:** 10.3390/foods14122089

**Published:** 2025-06-13

**Authors:** Mengxia Wu, Qin Xu, Han Ding, Dumin Zhao, Ying Wang, Baocai Xu

**Affiliations:** 1 School of Food and Bioengineering, Hefei University of Technology, Hefei 230601, China; w908956515@163.com (M.W.); qianxu70707@163.com (Q.X.); zdm00906@163.com (D.Z.); 2 Academic Publishing Center, Anhui University of Science and Technology, Huainan 232001, China; listitch@163.com; 3 Engineering Research Center of Agricultural Biotechnology, Ministry of Education, Hefei 230601, China

**Keywords:** natural antifreeze substances, artificial synthesis analogs, antifreeze mechanism

## Abstract

Freezing is a crucial technology for preserving food quality and extending shelf life. However, frozen storage often leads to protein oxidation, degradation, and cellular structural damage, compromising food palatability. To address these challenges, antifreeze substances have emerged as a promising solution. This review comprehensively summarizes the current research on antifreeze substances, including natural compounds and artificial analogs. For natural antifreeze substances, the mechanisms of antifreeze proteins (AFPs), antifreeze peptides (AFPPs), antifreeze polysaccharides (AFPLs), and antifreeze phosphates (AFPSs) are elucidated. Additionally, the preparation of artificial synthesis analogs and the application of antifreeze substances are discussed. By presenting their properties and research advancements, this review aims to provide a reference for the practical utilization of antifreeze substances in food-freezing applications.

## 1. Introduction

Fresh food is inherently perishable and fragile, rendering it vulnerable to mechanical damage and susceptible to attack by spoilage organisms [1]. To solve this problem, freezing is a widely adopted technique to mitigate spoilage and maintain food quality. However, in the storage process of frozen products, the formation and growth of ice crystals cause freezing damage to the products, which is closely related to the morphology, size, quantity, and distribution of ice crystals [2,3]. Excessive or uneven ice crystals lead to increased juice loss and decreased nutritional value during thawing [4]. Ice crystal instability or recrystallization changes the water distribution in food, resulting in the quality deterioration of frozen food. On account of the realities explained above, there exists a big challenge for the food manufacturers: how to develop appropriate technologies that can inhibit ice crystals, especially recrystallization, to preserve the structural and sensory characteristics of foods through the freezing process [5].

With advancements in freezing technologies, the concept of antifreeze agents has gained interest in the food industry. Antifreeze agents are substances added to liquid or food systems as additives to lower freezing points, enhance freeze resistance, and maintain product quality during frozen storage [6]. Generally, they are classified into two common species: natural antifreeze agents and artificial synthesis analogs [7]. Although studies have shown that some antifreeze agents (e.g., sucrose and fish/plant/insect-derived antifreeze proteins) perform well in freezing protection, systematic investigations into the mechanisms of different-source antifreeze substances and their specific effects in food systems are still needed.

**Figure 1:**
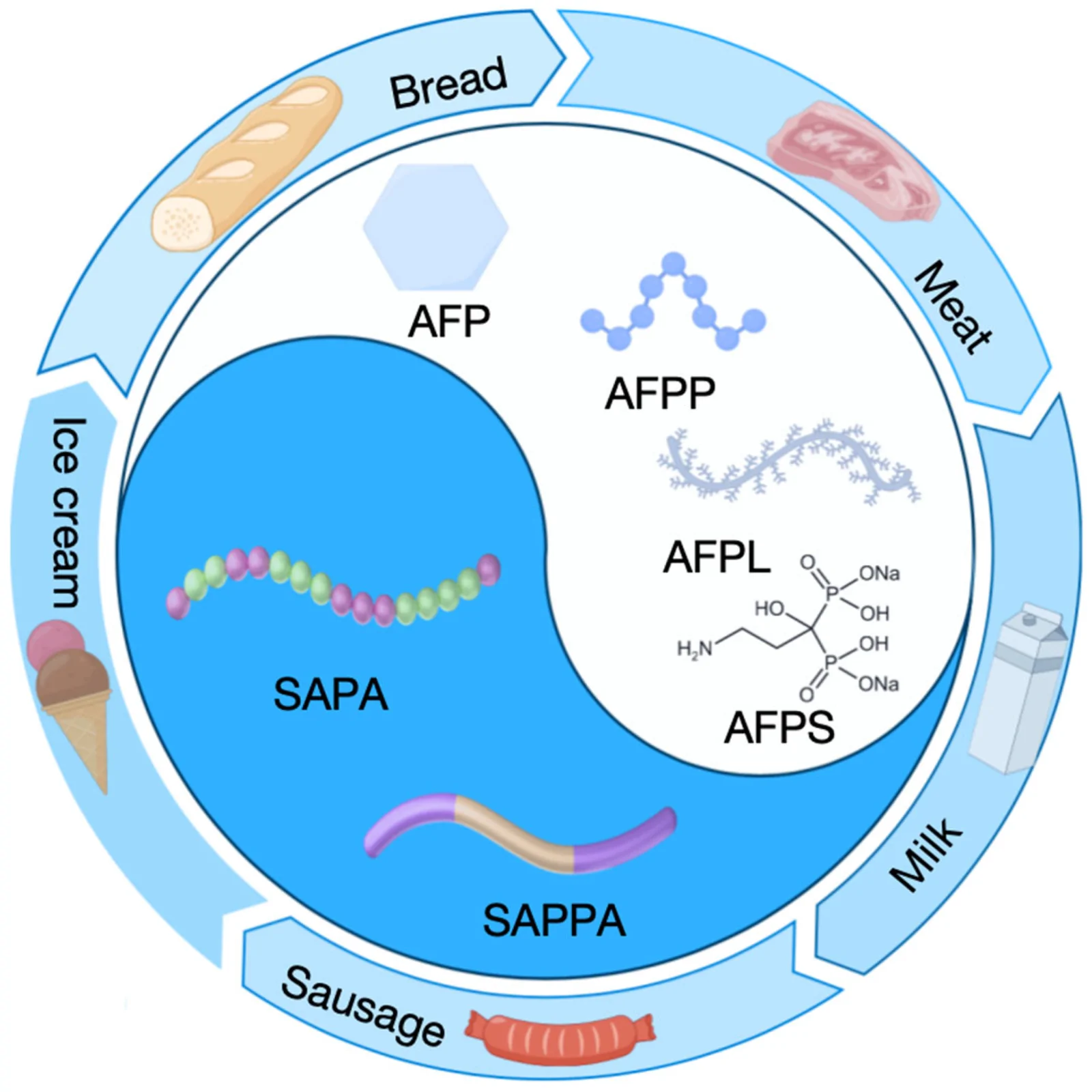
Classification and application of antifreeze substances. Antifreeze proteins (AFPs), antifreeze peptides (AFPPs), antifreeze polysaccharides (AFPLs), and antifreeze phosphates (AFPSs) and Antifreeze Proteins (SAPAs) and Synthetic Antifreeze Peptides (SAPPAs).

Herein, this review summarizes antifreeze substances (natural antifreeze agents and artificial synthesis analogs) and their application (Figure 1). It first describes natural substances, including antifreeze proteins (AFPs), antifreeze peptides (AFPPs), antifreeze polysaccharides (AFPLs), and antifreeze phosphates (AFPSs) and their mechanisms, followed by the preparation of two synthetic analogues, Synthetic Antifreeze Proteins (SAPAs) and Synthetic Antifreeze Peptides (SAPPAs). Meanwhile, the application of antifreeze substances is reviewed. Afterward, we emphasize that antifreeze agents have restrictions and challenges in many facets. In addition, the development trend of antifreeze in the future is discussed, and reasonable suggestions for the further study of freezing damage are put forward.

## 2. Natural Antifreeze Substance

Natural food antifreeze substances (AFP, AFPP, and derivatives like APFL and AFPS) employ distinct mechanisms to retard ice recrystallization and enhance moisture retention, thereby safeguarding the quality of frozen food products.

### 2.1. Mechanisms of Antifreeze Proteins (AFPs)

AFPs are functional proteins that specifically inhibit ice crystal growth and recrystallization [8]. Initially identified in Antarctic fish in 1969, they have since been found in diverse organisms, including insects, plants, bacteria, fungi, microalgae, and crustaceans [9]. Traditional freezing methods often generate large ice crystals in the food matrices, damaging tissue structures and compromising quality [10]. The incorporation of AFPs effectively suppresses ice crystal growth and recrystallization, offering broad application potential in the frozen food industry [11]. Their antifreeze activity stems from three distinct mechanisms.

#### 2.1.1. Thermal Hysteresis Activity (THA)

**Figure 2:**
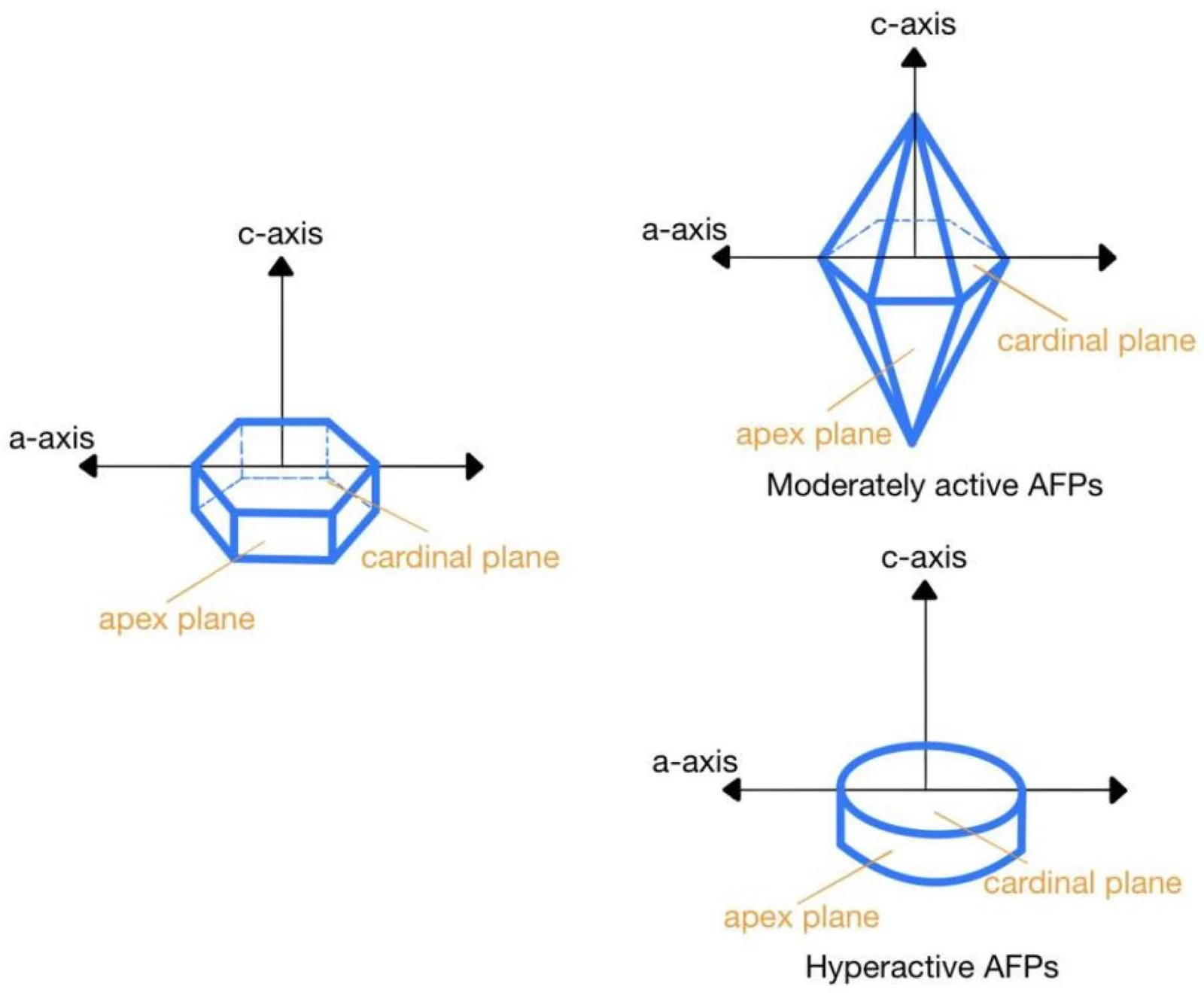
Schematic diagram of ice crystal growth direction.

AFPs lower the freezing point of water without altering its melting point, creating a difference between freezing and melting temperatures to enhance cryoprotection [12]. Based on their THA characteristics, AFPs are categorized into moderately active and hyperactive types [13]. The ice crystal growth modulation mechanism of these proteins is illustrated in Figure 2. Moderately active AFPs promote ice crystal growth parallel to the c-axis, exposing the basal surface to form hexagonal bipyramidal crystals with observable “growth bursts.” In contrast, hyperactive AFPs induce a-axis growth at a rate ~100 times faster than c-axis growth, forming cylindrical crystals [14,15].

#### 2.1.2. Ice Recrystallization Inhibition (IRI)

Ice recrystallization refers to the mass redistribution between ice crystals, where large crystals grow at the expense of smaller ones. This phenomenon can cause significant damage to the food matrices [16]. AFPs inhibit both the expansion of existing crystals and recrystallization, maintaining a uniform crystal size during freezing.

#### 2.1.3. Adsorption Inhibition

The inhibitory effect of AFPs on ice crystal growth is primarily mediated through protein adsorption. When AFPs are irreversibly adsorbed to the ice crystal surface, the crystallization of the surface covered by adsorption is inhibited, while the uncovered areas continue to crystallize, forming curved surfaces. This generates ice crystals with curved interfaces, increasing surface curvature and thereby lowering the freezing point of the surrounding solution via the Kelvin effect [17].

### 2.2. Mechanisms of Antifreeze Peptides (AFPPs)

AFPPs, including collagen polypeptides, are small-molecular-weight proteins that inhibit ice crystal growth. They reduce the freezing point of solutions through a non-colligative mechanism, leaving melting points unaffected [18]. Derived from food-grade proteins (e.g., edible gelatin, animal skin, fish scales) via controlled acid, alkali, or enzymatic hydrolysis, AFPPs are further separated by chromatography and ultrafiltration based on molecular weight [19,20]. The optimized peptide fractions exhibit multiple bioactivities, enhancing raw material utilization and promoting sustainable resource management. As such, they hold potential as replacements for conventional sugar-based cryoprotectants [21].

Numerous studies have demonstrated that the addition of hydrocolloids (e.g., gums and gelatins) significantly suppresses ice crystal growth in food systems [22]. Gelatin peptides inhibit ice crystal growth through mechanisms analogous to AFP/AFGP. Damodaran et al. [23] proposed a three-step IRI mechanism for fish gelatin hydrolysate peptides: (i) Non-specific electrostatic binding to negatively charged ice surfaces; (ii) structural rearrangement to enhance hydrogen bonding; (iii) the formation of hydrophobically stabilized complexes via adjacent nonpolar residues. Similarly, chicken collagen hydrolysate (CCH) reduces ice crystal growth in actomyosin systems, alleviating protein denaturation and oxidation while improving post-thaw solubility and digestibility [24].

### 2.3. Mechanisms of Antifreeze Polysaccharides (AFPLs)

The low-temperature environment presents challenges for organisms and food preservation. AFPLs are widely applied in frozen aquatic products to address low-temperature stresses by inhibiting ice crystal formation through multiple pathways [25]. Plant- and microorganism-derived hydrocolloids (e.g., κ-carrageenan, chitosan, locust bean gum) and polysaccharides function as functional fat substitutes in low-fat meats, enhancing water retention and texture [26,27]. AFPLs inhibit ice crystal formation and growth through diverse mechanisms, thereby safeguarding biological cells and food structures. With advancements in AFPL research, these compounds have shown broad application prospects in food preservation, biomedicine, and other domains.

The mechanisms can be explained through the following two aspects: (i) **Protein stabilization:** Sugar or sugar alcohol molecules bind to protein active groups via hydrogen bonds or ion interactions, creating steric hindrance to prevent protein aggregation and conformational change. Simultaneously, their polyhydroxyl structures chelate water molecules, promoting the conversion of free water to bound water and lowering the system’s eutectic point temperature to delay ice-induced protein damage. (ii) **Cryoprotection:** Low-molecular-weight sugars (e.g., glucose, chito-oligosaccharides, lactose) maintain natural protein conformation by competitively binding to surface water, whereas high-molecular-weight polysaccharides (e.g., xanthan gum and modified starch) form glassy matrices or induce α-helix structures to enhance freeze resistance [28].

### 2.4. Mechanisms of Antifreeze Phosphates (AFPSs)

AFPSs are widely used in aquatic products to enhance water retention and reduce drip loss, primarily including sodium tripolyphosphate (STPP), sodium hexametaphosphate (HMP), sodium tripolyphosphate (STP), tetrasodium pyrophosphate (TSPP), and sodium pyrophosphate (SPP) [29]. Although the effect of AFPSs on inhibiting protein freezing denaturation is not as strong as that of AFPLs, they significantly improve the water retention of the product. Consequently, they are often used synergistically with AFPLs, which exhibit stronger antifreeze effects, thereby greatly enhancing the overall antifreeze performance [30].

The mechanisms of AFPSs involve preventing freezing denaturation by altering the microenvironment of proteins, primarily through the following three pathways: (i) **Increasing ionic strength**: Upon phosphate addition, phosphate groups expose hydrophobic groups hidden inside the protein to its surface, thereby increasing ionic strength. During processing and storage, elevated ionic strength enhances the solubility of myofibrillar protein, improving water retention. (ii) **Buffering pH**: Phosphate solutions buffer the pH of the product, stabilizing it within an optimal range. Under these conditions, proteins are less prone to denaturation and maintain strong water-holding capacity. (iii) **Dissociating actomyosin**: Phosphate addition dissociates actomyosin into actin and myosin. Myosin, in particular, enhances meat water retention, thereby improving the quality of low-temperature meat products.

Different kinds of phosphates have different antifreezing mechanisms and are widely used in the production and transportation of aquatic products because of their excellent effect in improving water retention. The application and antifreeze mechanism of phosphate in different kinds of aquatic products are shown in Table 1.

## 3. Artificial Synthesis Analogs

Due to limited natural sources, complex production, and high costs, natural antifreeze agents cannot be mass-produced industrially, creating an urgent demand for new, easily obtainable, and low-cost antifreeze substances in the food industry.

### 3.1. Preparation and Application of Synthetic Antifreeze Proteins (SAPAs)

SAPAs are bioengineered mimics of natural antifreeze proteins or chemically synthesized polymers.

#### 3.1.1. Chemical Methods

Chemical methods are characterized by short reaction times, minimal equipment requirements, and straightforward processes. In this approach, natural antifreeze proteins are polymerized or cross-linked to form freeze-resistant gels.

For instance, Yang et al. [36] prepared a soybean protein-based gel electrolyte material using soybean protein isolate (SPI), acrylamide (AAm), and zinc chloride (ZnCl_2_) as raw materials. This material exhibited excellent freezing resistance. Wang et al. [37] incorporated natural fish antifreeze proteins into a hydrogel system consisting of chemically cross-linked polymers (acrylamide/sodium methacrylate) and physically cross-linked polyvinyl alcohol to develop a tunable antifreeze and biocompatible hydrogel sensor. Fu et al. [38] designed an agar/polyacrylamide (Agar/PAAm) dual-network hydrogel (Na-Agar/PAAM-AFP) containing AFP and sodium chloride (NaCl), which demonstrated good freezing resistance at −20 °C. However, SAPAs pose food safety concerns due to potential chemical residues.

#### 3.1.2. Genetic Engineering Expression

Genetic engineering enables the introduction of target genes into host cells to express recombinant proteins, thereby altering the genetic traits of organisms to produce novel varieties or products. Balamurugan et al. isolated the AFP gene from *Lolium perenne* and expressed it in tomato plants, which remained healthy under low-temperature stress. It was proved that the *Lolium perenne* AFP gene conferred freezing resistance in transgenic tomato [39]. Through the transcriptional regulation of the AFP gene, studies found improved cryopreservation efficiency in potatoes [40]. The recombinant production of AFP offers an economical alternative source offering different TH activities, unrestricted by season or natural sources [41]. Artificially synthesized recombinant antifreeze proteins (rAFPs) are generally designed from known natural AFPs. Compared to water, rAFPs significantly depress the supercooling point of solutions.

A recent comparative study investigated the effects of adding three rAFPs—*Daucus carota* antifreeze protein (rCaAFP), type II antifreeze protein from *Epinephelus coioides* (rFiAFP), and *Tenebrio molitor* antifreeze protein (rTmAFP)—on hydrated gluten, gliadin, and glutenin during freezing. Results indicated that these rAFPs, especially rTmAFP, provided superior cryoprotection for hydrated gluten matrices [42]. Yeh et al. [43] employed *Lactococcus lactis*, a food-grade microorganism of commercial significance, to secrete a novel recombinant type I AFP analogue (rAFP). Adding rAFP to frozen meat reduced drip loss and protein loss and improved juiciness in sensory evaluation. Similarly, rAFP-treated frozen dough exhibited enhanced fermentation capacity compared to untreated controls. Genetically engineered SAPA faces challenges of low expression levels and activity loss. Although the prokaryotic expression system achieves high yields, it tends to form inclusion bodies, resulting in the reduced activity of expression products or even loss.

#### 3.1.3. Recombinant Expression

Recombinant expression technology enables SAPA production via gene recombination, utilizing bacteria, fungi, animal, or plant cells as hosts for foreign gene expression [44]. Psychrophilic diatoms are abundant in polar sea ice. Uhlig et al. [45] identified *Fragilariopsis cylindrus*, a polar diatom carrying multiple AFP isoforms. Researchers expressed two heterogeneous AFPs from *F. cylindrus* in *Escherichia coli*; proteins refolded from inclusion bodies exhibited functional activity in crystal deformation, recrystallization inhibition, and thermal hysteresis.

Insect antifreeze proteins are renowned for their hyperactive THA. Lee et al. [46] isolated a codon-optimized ice-binding protein (LeIBP) from *Leucosporidium* sp. and achieved high-level expression in the methylotrophic *Pichia pastoris* system. Results indicated high-yield recombinant IBP production in pilot-scale fermentation, with rLeIBP secretion remaining stable and active over 6 days, with a THA of ~0.42 °C. Some plants in high altitudes usually need to adapt to a cold temperature environment, and they can produce AFPs to protect different tissue, e.g., spruce. Zhang et al. [47] identified four different spruce genes: PicW1, PicW2, PicM, and Pick. Sequence alignment revealed base substitutions/deficiency mutations with 97.61–99.25% identity. When expressed in *E. coli* and induced by IPTG (isopropyl-β-d-thiogalactoside), purified proteins exhibited THA values of 0.77 °C (PicW1), 0.78 °C (PicW2), 0.72 °C (PicM), and 0.86 °C (PicK), respectively—significantly higher than the control (BSA, 0.05 °C)—classifying them as hyperactive AFPs [48]. Antarctic yeast Glaciozyma antarctica secretes a ~27 kDa glycosylated ice-binding protein (GmAFP), which reduces drip loss and preserves texture in frozen fruits/vegetables [49].

In general, SAPA production predominantly uses engineered bacteria, raising persistent food safety concerns. A key challenge is mitigating risks associated with genetically modified hosts.

### 3.2. Preparation and Application of Synthetic Antifreeze Peptides (SAPPAs)

The application of AFPP remains constrained by limitations associated with conventional extraction from natural cryogenic organisms, including labor-intensive processes, low yields, and high costs. To address these challenges, recent studies have increasingly utilized genetic engineering for the heterologous expression of AFPs in microbial systems. For example, Uhlig et al. [45] demonstrated the production of *Bacillus cylindricus* AFPP analogs via *E. coli* heterologous expression. Chen et al. [50] reported enhanced survival rates, acid production, freeze stability, and metabolic activity in *Streptococcus thermophilus* following treatment with recombinant snow flea AFP (rsfAFP), supporting its potential as a cryoprotectant in frozen food applications.

Chemical synthesis is a common method for preparing polypeptides with specific amino acid sequences. Although recombinant protein technology enables the large-scale production of SAPPAs, chemical synthesis produces only small quantities of pure samples. Nevertheless, it remains valuable for producing peptides with precise amino acid sequences. *Dendroides canadensis* (Canadian moth larvae) AFPs (DcAFPs) were modified using solid-phase synthesis to create truncated analogs (DCR13, DCR26, DCR39) with varying lengths (13, 26, or 39 residues) [51]. Additional cyclic analogs (DCR26 cyclic and DCR39 cyclic) were synthesized by substituting native disulfide bridges with lactam linkages. Notably, this study was the first to demonstrate ice crystal growth inhibition by short, truncated DcAFP analogs, enhanced further by citrate supplementation. Subsequent work by Yang et al. [52] and Zhang et al. [53] yielded synthetically designed AFPPs with sequences GAGP[(GVGVP)(GEGVP)_9_]_2_GWPH_6_ and DTASDAFAAAAL, respectively, exhibiting robust recrystallization inhibition and THA.

Emerging strategies involve the glycosylation of fish collagen peptides to develop eco-friendly AFPP alternatives for frozen foods. Chen et al. [54] showed that non-enzymatically glycosylated SAPPAs preserved cellular integrity and improved *Streptococcus thermophilus* viability, suggesting their utility as novel cryoprotectants. Liu et al. [55] further demonstrated antifreeze activity in tilapia via the transglutaminase-catalyzed glycosylation of fish collagen peptides with glucosamine hydrochloride.

Furthermore, while synthetic antifreeze agents are effective, they are typically produced using genetically engineered bacteria, which carry toxicological risks (e.g., potential carcinogenicity), necessitating rigorous safety evaluations. Mitigating risks from engineered bacteria remains a critical challenge. Despite promising applications in cryopreservation, cryogenic transport, and tissue engineering, industrially viable synthetic antifreeze substances remain underdeveloped. Key knowledge gaps regarding their mechanisms of action and structure–function relationships persist, requiring further research to realize their full potential in the frozen food industry.

## 4. Application and Limitations of Antifreeze Substances

### 4.1. Application and Limitations of AFPs

AFPs are effective in altering ice crystal morphology, inhibiting recrystallization, and stabilizing cell membranes [8]. These proteins are widely used as cryoprotectants in the food industry, including aquatic products preservation, dairy processing, dough and bakery products, and meat products. Adding AFPs to frozen aquatic products significantly reduces ice crystal damage to fish muscle tissue, maintains cell membrane integrity, reduces drip loss rates after thawing, and better preserves texture and flavor. Cheng et al. [56] verified the protective effect of AFP III on the cod myofibrillar structure using low-field nuclear magnetic resonance (LF-NMR) and Fourier transform infrared spectroscopy (FTIR), achieving a 42% reduction in drip loss.

In ice cream production, the size and distribution of ice crystals are critical for determining taste. AFP supplementation reduces average ice crystal size and enhances texture [57]. The repeated freezing of dough leads to gluten structure damage, texture hardening, and a decline in fermentation efficiency. Studies demonstrate that adding carrot-derived AFPs (*Daucus carota*) to dough improves fermentation capacity, reduces freeze–thaw water loss, and maintains the storage quality and ultrastructure of the dough [42,58]. Meat products are particularly susceptible to ice crystal damage during processing and storage, as repeated freeze–thaw cycles exacerbate tissue damage and juice loss. AFPs effectively mitigate freezing-induced damage in meat products, maintain their original tissue structure, reduce drip loss, and maximize nutrient and flavor retention [59]. Tang et al. demonstrated the cryoprotective effect of krill-derived AFPs on *Lactobacillus bulgaricus*, which could be applied to the frozen storage of fruits and vegetables [60].

For AFPs, structural conformation, molecular weight, and ambient temperature significantly affect their ice inhibition activity. Compared with disaccharides (e.g., lactose, sucrose) and hexose (e.g., glucose and fructose), pentose (e.g., ribose and xylose) exhibits poorer freezing resistance. This difference arises because abundant active aldehyde groups in pentoses react with amino groups, promoting protein molecule aggregation [61]. Limited natural AFP sources hinder their widespread application in the food industry. Therefore, while ensuring safety and reducing large-scale production costs, deepening mechanistic understanding is essential [11].

### 4.2. Application and Limitations of AFPPs

Recent studies have highlighted the cryoprotective potential of gelatin-derived compounds. Wang et al. [62] demonstrated that supplementing *Lactobacillus bulgaricus* with collagen hydrolysate during freezing significantly enhanced bacterial survival rates, further substantiating gelatin’s utility as a cryoprotective agent. Kittiphattanabawon et al. [63] incorporated gelatin hydrolysate from Japanese scombroid skin into a freeze–thawed shrimp model system and observed that this hydrolysate exhibited comparable efficacy to mixed phosphates in retarding lipid oxidation. This suggests its potential to mitigate quality deterioration in frozen shrimp by inhibiting oxidative processes. More recently, AFPPs derived from fish skin gelatin—extracted via hot-water extraction and purified through multi-step chromatography—were shown to markedly reduce freezing-induced damage in *Escherichia coli* and improve post-thaw survival rates [64]. These findings underscore the antifreeze activity of gelatin-derived peptides and their promising applications in cryopreservation, while providing a theoretical foundation for the further functional exploration of gelatin polypeptides.

Yet, AFPPs suffer from a bitter taste and poor color, affecting product quality. Challenges in separating, purifying, and identifying novel AFPPs persist, requiring deeper investigations into their fundamental functions [65].

### 4.3. Application and Limitations of AFPLs

Over the past decade, research has demonstrated the significant potential of sugar- and sugar alcohol-based cryoprotectants in food preservation and microbial cryopreservation. Trehalose, for example, is a carbohydrate present in living organisms whose non-reducing disaccharide structure (α-D-glucopyranosyl-(1→1)-α-D-Glucopyranoside) enables key anti-stress protection in anhydrobiotic organisms [66]. It is well established that proteins and cell membranes can be inactivated or denatured under external stress conditions (e.g., drying, dehydration, high temperature, low temperature, and oxidation). Trehalose can effectively protect them from these hazards and is therefore of significant importance in biotechnology. Wu et al. [67] found that adding trehalose to myofibrillar protein from *Lateolabrax japonicas* and freezing it at −18 °C for 90 days significantly increased the contents of mercapto groups and unfrozen water in myofibrillar proteins compared with the control group (*p* < 0.05). Zhang et al. [68] demonstrated that trehalose–alginate oligosaccharide synergy reduces thawing and cooking losses in frozen shrimp.

Recent advancements in polysaccharide cryoprotectants have expanded the field of food cryopreservation. Xylose–mannan (APS-A), developed by Sun et al. [69], exhibits remarkable thermal stability, the shear-thinning properties of non-Newtonian fluids, and exceptional freeze and oxidation resistance. Guerreiro et al. [70] extracted exopolysaccharide Fococol from the *Enterobacter* A47 strain, which displays shear-dilution and polyanion properties. These properties promote interactions between the polysaccharide and water–ice interfaces, ultimately hindering ice crystal growth. Cassava-modified starch has good water absorption, promotes the conversion of free water to bound water, thereby reducing water migration and inhibiting ice crystal recrystallization, and shows a significant texture improvement effect in low-temperature meat products [71,72,73]. Qin et al. [74] showed that corn-modified starch significantly reduced cooking and freeze–thaw losses in beef gel (*p* < 0.05), likely via starch–protein complex formation.

Although AFPLs have shown broad application prospects in many fields, they still face several challenges. While AFPLs provide excellent antifreeze properties, they also introduce moderate sweetness and caloric content to products, rendering them unsuitable for patients with hyperglycemia or hyperlipidemia. The high production costs of certain antifreeze sugars limit their large-scale adoption. Additionally, the safety assessment of novel antifreeze sugars remains incomplete, necessitating further studies on their impacts on human health and the environment. Although the mechanisms of AFPLs are partially understood, further investigations into their specific action pathways and regulatory mechanisms are still required.

### 4.4. Application and Limitations of AFPSs

Rattanasatheirn et al. [75] soaked pacific white shrimp chilled for 7 days in a 2.5% NaCl solution containing 0.875% SAPP and 2.625% TSPP for 2 h, observing increased transparency and reduced cooking loss. This indicates that phosphate-treated frozen shrimp exhibit enhanced water retention capacity. Upon the phosphate addition, protein surface hydrophobicity increases, and water molecules may tightly bind to phosphate or proteins via ionic interactions. Three shellfish species—oyster, scallop, and clam—were respectively soaked in complex sodium phosphate solutions and freeze-dried. Results showed that the rehydration capacity first increased and then decreased with concentration, likely due to elevated osmotic pressure causing shellfish meat dehydration [35]. Lee et al. [76] investigated the effects of different phosphates on the frozen quality of Alaskan cod fillets and surimi. They found that STPP and TSPP more effectively inhibited trimethylamine-N-oxide demethylase (TMAOase) activity than other phosphates, possibly by altering the redox potential states of cofactors. Moreover, STPP and TSPP exhibited strong affinity for TMAOase, enhancing salt-soluble protein extraction. To improve aquatic product water retention, it can be combined with phosphates. Zhang et al. [77] studied the antifreeze effects of soaking shrimp with different proportions of complex phosphate, trehalose, and sorbitol antifreeze agents, and the results showed that the combination of antifreeze agents significantly improved the water-binding ability of shrimp. The mechanism of antifreeze was analyzed. It may be that the phosphorylated trehalose regulates the pH value of the shrimp protein system, which makes the protein stable and not susceptible to degeneration. Wu et al. [78] added a certain proportion of trehalose, polydextrose, and xanthan gum into steaks, and found that the composite antifreeze agent could significantly reduce the loss rate, juice loss rate, cooking loss rate, and TBARS values of prepared steaks during freezing storage while maintaining their color and organizational structure.

AFPS effectiveness depends on solubility, antioxidant performance, and dosage. Critically, SPP and STPP degrade under freezing conditions. AFPS intake may also exacerbate hypertension and chronic kidney disease symptoms [32]. A comparison of the application and limitations of several antifreeze substances is shown in Table 2.

## 5. Conclusions and Prospects

This review provides a comprehensive overview of antifreeze substances, including their mechanisms, applications, and challenges. Reasonable suggestions are put forward for further studies on improving the quality of low-temperature food. The sources of natural antifreeze are limited, and the production cost is high. Synthetic analogs offer promise but require clearer mechanistic understanding and safety validation. There may be other undiscovered protein or non-protein molecules with antifreeze activity in animals, plants, and microorganisms, which need further research and exploration. Additionally, the development of efficient, green, and low-cost production (i.e., treatment, separation, and purification) of antifreeze substances is crucial, with broad market demand and application prospects.

## Figures and Tables

**Table 1:** Application and antifreezing mechanism of phosphate in different kinds of aquatic products.

Type	Common Phosphate	Function	Application	Reference
Fish	STPP, SPP, HMP	Increase fish soaking weight gain rate and reduce cooking loss	Tilapia, catfish, snapper	[31,32,33]
Shrimps	STPP, SPP, compound phosphate	Prevent frozen degeneration of shrimp meat and increase water retention	South American white shrimp, *Penaeus vannamei*	[34]
Molluscs	STPP, SPP, compound phosphate	Keep the moisture in the shell meat and improve the edible quality	Oysters, scallops, flower clams	[35]

**Table 2:** Comparison of the application and limitations of antifreeze substances.

Type	Application	Function	Limitations
AFP	Preservation of aquatic products. Improve the texture of ice cream. Maintain the fermentation capacity of frozen dough. Reduce frostbite from meat products.	Inhibition of ice crystal growth/recrystallization. Stable cell membrane. Reduced SAP loss. Maintaining protein structure.	1. Activity is affected by structure/molecular weight/temperature. 2. Pentose (ribose, etc.) has poor freezing resistance and easily causes protein aggregation. 3. Natural sources are limited and large-scale application costs are high.
AFPP	Microbial cryoprotection. Inhibition of lipid oxidation of frozen shrimp. To improve the survival rate of *E. coli* after freezing.	Collagen hydrolysate enhanced the freezing resistance of the strain. Good antioxidant effect. Reduce the freezing damage of bacteria.	1. Bitter taste and color adversely affect product quality. 2. High technical difficulty in separation and purification. 3. The underlying functional mechanisms need to be further studied.
AFPL	Reducing thawing/cooking losses of seafood such as shrimp. Improve the texture of low-temperature meat products. Stabilize protein/cell membranes as a bioprotective agent.	Trehalose and alginate oligosaccharides synergetic for water retention. APS-A showed thermal stability/freezing resistance. The modified starch inhibited the recrystallization of ice crystals.	1. Introduce sweetness and heat, not suitable for patients with high blood sugar/high blood lipid. 2. High production cost. 3. Insufficient safety evaluation of novel polysaccharides. 4. The mechanism of action is unclear.
AFPS	Improve the transparency of shrimp and reduce cooking loss. Optimize the rehydration of freeze-dried shellfish. Defrosting of cod/surimi.	Phosphate enhances ion repulsion. STPP/TSPP inhibited enzyme activity and promoted salt-soluble protein extraction. Complex sugars enhance water retention.	1. SPP/STPP is easily degraded in freezing. 2. Increased risk of hypertension/chronic kidney disease. 3. Efficacy is limited by solubility/antioxidant/dosage.

## Data Availability

No new data were created or analyzed in this study. Data sharing is not applicable to this article.
